# Mortality rate, carbon emissions, renewable energy and per capita income nexus in Sub-Saharan Africa

**DOI:** 10.1371/journal.pone.0274447

**Published:** 2022-09-15

**Authors:** Bosede Ngozi Adeleye, Aminat Olayinka Olohunlana, Cleopatra Oluseye Ibukun, Titilayo Soremi, Barnabas Suleiman

**Affiliations:** 1 Department of Economics and Development Studies, Covenant University, Ota, Nigeria; 2 Centre for Economic Policy and Development Research (CEPDeR), Covenant University, Ota, Nigeria; 3 Regional Centre of Expertise (RCE) Ogun, Covenant University, Ota, Nigeria; 4 Department of Economics, University of Lagos, Lagos, Nigeria; 5 Department of Economics, Obafemi Awolowo University, Ife, Nigeria; 6 Department of Political Science, University of Toronto Scarborough, Ontario, Canada; 7 Department of Sociology, Baze University, Abuja, Nigeria; Shenzhen University, CHINA

## Abstract

This study exclusively contributes to the health-environment discourse by using mortality rates, carbon emissions (proxy for environmental degradation), renewable energy and real per capita income to investigate these intrinsic relationships. This study uses an unbalanced sample of 47 Sub-Saharan African countries from 2005–2019 to reveal that: (1) both carbon emissions and renewable energy are associated with higher mortality rates; (2) real per capita income is associated with reducing mortality rates; (3) per capita income attenuates the effect of renewable energy on mortality rates, (4) persistency in mortalities exist; and (5) the health-environment-energy-income dynamics differ across income groups. Additionally, this study submits that the interaction of renewable energy and real per capita income dampens the positive effect of renewable energy on mortality rates and supports the argument that income levels lessen the extent of mortalities. Besides, these results vividly show that real per capita income reduces the devastating effect of renewable energy on infant and under-5 mortality rates from 0.942% to 0.09%, 2.42% to 0.55%, 1.04% to 0.09% and 2.8% to 0.64% for high and middle-income countries, respectively. This is a novel and significant contribution to the health-environment literature. Hence, real per capita income is a crucial determinant of mortality rate. Policy recommendations are discussed.

## 1. Introduction

To *“ensure healthy lives and promote well-being for all at all ages”* and *“make cities and human settlements inclusive*, *safe*, *resilient and sustainable”*, this study aligns with the 2030 United Nations Sustainable Development Goals 3 and 11 to examine the health-environment-energy-income paradox by documenting empirical discoveries which fill a lacuna in the literature. One of the most utilized child health indicators are infant and under-5 mortality rates [1–3]. The rate of infant mortality in a locality highlights not only the quality of healthcare available to the child, but also the level of healthcare affordable by the family or guardians of the child. While the quality of healthcare can be dependent on the health system that is prevalent in the country, the affordability can be dependent on the level of economic opportunities that are accessible in the country. Sub-Saharan Africa (SSA) holds the promise of supporting a teeming population and contributing to technological and economic advancement in the world [4, 5]. Albeit the region is beleaguered with multifaceted challenges from economic to political issues. With a rapidly growing population, a major concern for governments in the region is the challenge of maintaining the health of the population, especially the health of children. To assess health outcomes, specific indicators such as the environment, access to clean energy, access to basic amenities, education and health awareness are relied upon to track progress and assess their influence on human health. Hence, this multi-dimensional investigation takes a new perspective and highlights findings on if carbon emissions, renewable energy and real per capita income reduce the incidence of infant and under-5 mortality rates in SSA. Conclusions reveal, *inter alia*, that carbon emission is associated with increased mortality rates. Though, renewable energy is positively associated with high mortality rates the interaction with real per capita income yields negative outcomes to attenuate the devastating effect of renewable energy on mortality rates. In essence, per capita income is a crucial determinant of mortality rates. These are significant contributions to the health-environment literature and provides the justification for engaging this study.

Studies have identified causes of infant mortalities in SSA. For example, Ewbank et al. [6] identified neonatal causes, diarrhoea, pneumonia, malaria, measles, meningitis, malnutrition, tuberculosis, as the main causes of infant mortality in some SSA countries. Also, Ester et al. [7] identified neonatal cause, malaria, diarrhoea, HIV/AIDS, measles, and accidents. The loss of infants due to preventable illnesses may be linked to healthcare services, and neonatal deaths can be linked to poor maternal health. Studies that examined macro level health determinants are Shobande [8], Chewe and Hangoma [9], Fayissa and Gutema [10], and Ogunleye [11]. Important factors identified to determine life expectancy and child mortality include income per capita, education, food availability, and alcohol consumption [10–12]. Based on this, we fill a gap in the literature by considering two factors, specifically environmental factors–carbon emissions and renewable energy to examine their significance as they pertain to the occurrence of infant mortality and under-5 mortality rates. Five questions are outlined: (i) does carbon emission contribute to infant and under-5 mortality rates in SSA? (ii) does renewable energy exert significant effect on infant and under-5 mortality rates and does the effect reduce or increase with the moderation of per capita income? (iii) does per capita income contribute significantly to infant and under-5 mortality rates? (iv) do these effects significantly differ by income groups? (v) is infant and under-5 mortality rate persistent in SSA? Static and dynamic estimation techniques are engaged to methodically probe these questions with an unbalanced panel data of eleven variables on 47 SSA countries obtained from World Bank [13] World Development Indicators from 2005 to 2019. The rest of the study is structured as follows: Section 2 discusses the empirical literature; Section 3 outlines the data and model; Section 4 interprets the results and Section 5 concludes with policy recommendations.

## 2. Literature review

### 2.1. Theoretical background

Based on the ability to explain the essence of human capital and the preservation of infant health, this study acclimates the theoretical basis of Grossman [14] demand for the healthcare model and Becker [15] human capital hypothesis. The fundamental philosophy underlying Grossman [14] is the utility function that features healthcare directly as a good in which households and individuals derive satisfaction while Becker [15] hypothesizes the importance of certain factors that increase the resourcefulness in people. Activities such as schooling, on-the-job training, medical care, migration and information search about prices and income altogether increase an individual’s propensity to earn and maintain a decent standard of living. Therefore, by taking the health dimension of human capital into consideration these hypotheses are sufficient to lay the empirical construct and modelling for this study.

### 2.2. Empirical review

Mortality is globally considered among the major health effects of air pollution and a welfare indicator. The reduction of mortality rate remains a fundamental Sustainable Development Goal because the factors that reduce or increase mortality rate are often socio-economic causes [16] such as education, access to clean water and other economic considerations [17, 18]. The extant literature on mortality rate have used proxies ranging from child to adult mortality or disease-specific mortality, but this study centres around infant and under-5 mortality and the review is sub-sectioned into three different strands.

#### 2.2.1. Mortality rate and carbon emissions

Over the past decades, considerable empirical attention has been given to the effect of carbon emissions on human health, both at the national and global levels. Earlier investigations from studies that focused on the United States [19, 20] suggest that reductions in carbon emissions significantly lead to a decline in mortality rate. A major uniqueness of Currie and Neidell [19] is the use of individual-level data and carbon monoxide (CO) as one of the proxies for pollutants while Jacobson [20] measured carbon emissions using carbon dioxide (CO_2_). Nonetheless, both studies show that reductions in carbon emissions will reduce mortality rate. Other country-specific studies include Arceo-Gomez et al. [21] whose study shows that an increase in carbon emission leads to a rise in Mexico’s infant mortality rate despite the evidence of a non-linear nexus. In addition, Sinha et al. [22] find that the relationship between India’s CO_2_ emissions and infant mortality is bidirectional while Sokadjo et al. [23] find a positively significant association between CO_2_ emissions and under-5 mortality in Benin Republic.

Using panel data approaches, Fotourechi [24] and Husnain et al. [25], examined the health effects of air pollutants in 60 developing countries and four developing South Asian countries (India, Bangladesh, Pakistan and Sir Lanka) respectively. Fotourechi [24] applied the recursive simultaneous equation approach on unbalanced data that covered between 1990 to 2010, while Husnain et al. [25] applied the fixed-effect method on data over the period 1978 to 2010. Irrespective of the methodological differences, their findings appear comparable. For the former study, the results suggest that a percentage increase in carbon emission leads to approximately 0.4% increase in the rate of infant mortality, while the outcome of the latter shows that a percentage increase in emissions result in approximately 0.5% rise in the rate of infant mortality.

Still in the context of panel studies, some scholars [8, 26, 27] applied the generalised method of moment (GMM) instrumental variables approach to account for possible endogeneity issues in the mortality-economic growth nexus. Aliyu and Ismail [26] assessed the influence of poor air quality on human mortality (child and adult) using data that comprised 35 African countries for the period 1995 and 2011. Shobande (2020) [8] pooled data from 1991–2014 across 23 African countries while Rasoulinezhad et al. [27] focused on the Commonwealth of Independent States (CIS). Nevertheless, the three studies establish a positive and significant nexus between carbon emissions and mortality rate.

#### 2.2.2. Mortality rate and renewable energy

Generally, SSA is considered the region with the largest proportion of individuals using pollutants for household energy and the region has the least access to renewable energy globally [28–30]. In view of this, Hanif [31] examined the relationship between energy consumption habits and health (measured by mortality rate) in 35 SSA countries. Alternatives to fossil fuels such as wind, solar, hydropower, biomass, and geothermal are becoming increasingly reliable and popular across the globe, particularly because they are fundamental to human and economic development. Unsurprisingly, recent empirical evidence from 155 countries [32] and 19 Latin America and Caribbean countries [33] provide evidence showing that the use of renewable energy reduces child mortality despite their methodological differences. Koengkan et al. [33] applied the panel quantile regression approach on data from 1990 to 2016 to show that the effect of renewable energy on mortality rate can be direct and indirect. Majeed et al. [32] accounted for endogeneity bias by using the system GMM approach and amongst other findings to show that the consumption of renewable energy has a reducing effect on mortality rate in the region. Conversely, Sene [34] deployed data on 54 African countries to find that access to renewable energy in the region has no direct correlation with socio-economic development indicators.

In the United States, Millstein et al. [35] analysed the benefits of the rapid rise in solar and wind energy usage between 2007 and 2015. Although the study notes that benefits from the use of these renewable energy sources vary dramatically over time and across regions. Given the widespread diversification of energy portfolio and the adoption of natural gas as a clean energy source and a means of limiting carbon emission, studies like Cesur et al. [36] examined its effect on infant mortality rate. An annual province-level data was employed and the findings of the study suggest that a percentage point increase in the intensity of natural gas leads to a 4% decrease in infant mortality. In agreement with this, recent studies on China [37, 38] provide evidence showing that the use of natural gas as a clean source of energy reduces China’s mortality rate, while another study of 20 developing Asian countries [39] shows that a percentage increase in the consumption of clean energy reduces mortality rate by 0.5% in the long-run.

#### 2.2.3. Mortality rate and economic growth

The question of whether mortality rates affect economic growth or vice versa has been extensively examined in the literature, albeit with mixed findings. While a considerable literature [40–42] proposes that a reduction in mortality rates promotes economic growth, Bhalotra [43], Erdoğan et al. [44] and Morgado [45] show that mortality rates of countries reduce as their real per capita GDP increases. Besides, evidence from Ensor et al. [46] and Alexander et al. [47] submits that mortality rates respond differently to economic changes, and Nishiyama [48] adds that the mortality-economic growth nexus is asymmetric in nature. That is, negative economic growth has a strong adverse effect on the mortality rate (infant), while positive economic growth has a weak and mixed reducing effect. Amiri and Gerdtham [49] applied the Granger causality and Data Envelopment Analysis (DEA) approach on a panel of 180 countries between 1990 and 2010 to find that the causal relationship between under-5 mortality and economic growth is bi-directional in 58% of the countries, 27% shows that it is the changes in under-5 mortality that affects economic growth, 8% suggests otherwise, while 7% of the countries established no relationship between under-5 mortality and economic growth. Afterwards, from a similar study of 175 countries, Amiri and Linden [50] found evidence suggestive of a more frequent causal effect of per capita GDP on child mortality in low-and lower-middle-income countries.

Applying the least squares approach, Demetriou and Tzitziris [51] shows that GDP per capita and infant mortality rate follow an increasing returns to scale. That is, at the highest level of income, infant mortality reduces until it reaches a point of its lowest value and then it starts to rise. Nevertheless, findings from Ray and Linden [52] through the GMM-2SLS approach reflect a negatively significant relationship between infant mortality rate and per capita GDP particularly in poor countries with less than 1000USD per capita income with similar outcomes from Jayadevan [53] who applied a structural equation model on a panel of 181 countries. Furthermore, Kammerlander and Schulze [54] focused on 46 developing countries using the household as the unit of the analysis. The study confirms that economic growth at the local level leads to a decline (moderate) in infant mortality. Overall, these studies expound the linear effects of carbon emissions, energy use and per capita income on infant and under-5 mortality rates. While most agree that economic growth alone cannot be relied upon to curb child mortality [43, 55] none examined any nonlinear interactions. Therefore, this current study extends the literature to fill the gap by probing whether the interaction of renewable energy with per capita income is associated with increasing or reducing the effect of renewable energy on mortality rate. In other words, we examine how renewable energy is associated with mortality rate at different levels of per capita income. This is because higher per capita income allows the government to invest more in public health facilities, which may reduce mortality rate.

## 3. Data and analytical approach

### 3.1. Data and model

This paper modifies the theoretical framework and modeling of Grossman [14], Becker [15], Shobande (2020) [8] and Barua et al (2022) [3] to specify human capital proxied by infant and under-5 mortality rates as a function of the environment, energy usage, economic and social factors. It uses an unbalanced panel data of eleven variables on 47 SSA countries (List of countries and income classification are displayed in S1 File) from 2005 to 2019 to investigate the effect of carbon emissions proxy *(CO2PC)*, renewable energy consumption (*REN*) and real gross domestic product per capita (*PC*) on infant mortality rate (*MINF*) and under-5 mortality rate (*MU5*). The control variables are female secondary school enrolment (*SECF*), health expenditure per capita (*HEXPC*), access to basic sanitation (*BSAN*), inflation rate measured by the index of consumer prices (*INFL*), urban population *(URB)*, and renewable electricity (*RELEC*) included for robustness checks. All variables are sourced from World Bank [13] World Development Indicators.

To address the study objectives a two-step model is constructed. Controlling for other variables, the first model sets up mortality rate (infant and under-5) as a function of carbon emissions, renewable energy consumption, and real per capita income. That is:

$$\ln Y_{i,t}=\alpha +\psi \ln CO2PC_{i,t}+\psi_{2}\ln REN_{i,t}+\psi_{3}\ln PC_{i,t}+\omega \ln Z{'}_{i,t}+d_{t}+K_{i}e_{i,t}$$ [1]

Where, *Y* represents each of the dependent variables *(MINF*, *MU5)*; *α* is the intercept of the model; ***Z’*** is the vector of control variables; *d*_*t*_ represents year dummies that capture variations of the dependent variable; ***K***_***i***_ is the income group (high, low and middle-income) dummy variable which equals 1 if the country is in that income group and 0, otherwise; ψ_*i*_ and ω are parameters to be estimated; *e* is the idiosyncratic error term assumed to be white noise. The two-way error component model is specified to recognize the distinct heterogeneities among the income groups. To control for outliers and establish an elasticity relationship, all variables with the exception of *INFL* are transformed into their natural logarithms.

The second model augments the initial model with the inclusion of an interaction term, ln*REN* * ln*PC* to (i) gauge if the initial effect of carbon emissions on mortality rate is sustained and (ii) whether per capita income lessens or boosts the *total effect* of renewable energy on mortality rates. The augmented model is specified as:

$$lnY_{i,t}=\xi +\gamma_{1}\text{ln}CO2PC_{i,t}+\gamma_{2}\text{ln}REN_{i,t}+\gamma_{3}\text{ln}PC_{i,t}+\gamma_{4}(\text{ln}REN_{i,t}*\text{ln}PC_{i,t})+\varphi \text{ln}X{'}_{i,t}+d_{t}+G_{i}+v_{i,t}$$ [2]

Where, the variables and symbols are analogous to those in Eq [1]. For dynamic analysis, Eqs [1] and [2] are modified with the inclusion of the lagged dependent variable as regressors to test for the persistency of mortality rate. Furthermore, to check if the outcomes of Eqs [1] and [2] differ by income group classification, the sample is divided into three (Note: Upper-middle and lower-middle income countries are classified as middle-income countries) distinct groups: (i) high income, (ii) middle income and (iii) low-income countries.

On *a priori* expectations, the effect of *CO2PC* on child mortality is expected to be positive since environmental hazards pose severe health risks to in-dwellers of affected regions, including infants and under-5 children. The effect of *REN* may be asymmetric. Positive, if renewable energy-enabled facilities are too expensive for nursing mothers to access such facilities causing deaths of infants and under-5 children. However, when proceeds from increased energy use is channelled towards the enhancement of human welfare, mortality rate is expected to fall, thus, indicating a negative relationship. Mortality rate may fall if renewable energy-enabled facilities are accessible at minimal cost to nursing mothers. Also, rising income (*PC*) is expected to exert a downward pressure on mortality rate as individuals are more disposed to accessing healthcare services and resources. Hence, a negative relationship between GDP per capita and child mortality rate is expected. Government spending on health per capita (*HEXPC*) is expected to have a negative relationship with child mortality rate since increased spending on health should expand existing capacities in the health sector which improves the health of infants and under-5 aged children. As female secondary education (*SECF*) improves, it is expected that child mortality rates drop since more knowledgeable mothers are expected to understand the essentials of childcare and upbringing better than those who hold little or no education. Higher commodity price (*INFL*) is expected to have severe effect on child mortality since inflated prices of goods dampen the ability of individuals to afford the basic essentials of living as cost-of-living rises. With a reduction in purchasing power, cost of healthcare becomes high, leading to increased possibilities of child deaths. Better sanitation (*BSAN*) is expected to reduce mortality rate while urbanization (*URB*) worsens mortality incidences since increased population rate over resources expansion stresses the available facilities and healthcare resources in urban centres. Renewable electricity *(REL)* is expected to show an indirect relationship with child mortality rates. Table 1 details the variables and expectations.

**Table 1:** Variables description and expectations.

Code	Description	Expectations
*MINF*	Mortality rate, infant (per 1,000 live births)	N/A
*MU5*	Mortality rate, under-5 (per 1,000 live births)	N/A
*CO2PC*	CO2 emissions (metric tons per capita)	+
*REN*	Renewable energy consumption (% of total final energy consumption)	+/-
*PC*	GDP per capita (constant 2010 US$)	-
*HEXPC*	Current health expenditure per capita (current US$)	-
*SECF*	School enrolment, secondary, female (% gross)	-
*INFL*	Inflation, consumer prices (annual %)	+
*BSAN*	People using at least basic sanitation services (% of population)	-
*URB*	Urban population (% of total population)	+
*REL*	Renewable electricity output (% of total electricity output)	-

Source: Authors’ Compilations from World Bank (2020) WDI

Following the analytical procedure of Adeleye et al. [56] and Adusei and Adeleye [57], the sign of the coefficient of the interaction term, γ_4_ evaluates if the interaction of *PC* on *REN* enhances or distorts the *total* effect of renewable energy consumption on mortality rate. The total effect of *REN* on mortality rate is computed as:

$$\frac{\partial \ln Y}{\partial \ln REN}=\gamma_{2}+\gamma_{4}\ln PC$$ [3]

However, since the expected coefficient of *REN* may be positive or negative, evaluating its total effect on mortality rate is not outrightly predictable. For instance, in the event that γ_2_ is positive, a positive (negative) γ_4_ indicates that *PC* amplifies (eases) the devastating effect of renewable energy consumption rates. Similarly, if γ_2_ is negative, a positive (negative) γ_4_ indicates that *PC reduces* (strengthens) the improving effect of renewable energy consumption on mortality rates. Finally, γ_4_ = 0 is an indication that the interaction of *PC* with *REN* has no significant effect on mortality rate.

### 3.2. Estimation techniques

To methodically draw the significance of carbon emissions, renewable energy and per capita income on mortality rate, the study adopts the use of static and dynamic models which serve as robustness for one another. Similar studies use these estimation approaches (Barua et al., 2022; Adeleye & Eboagu, 2019; Adeleye & Jamal, 2020; Adeleye, Osabuohien, & Bowale, 2017; Shobande, 2020) [3, 8, 58–60]. The static technique is the bootstrapped least squares dummy variables (*BLSDV*) technique, also known as "fixed effects" that account for heterogeneities across the panels using dummy variables. The dynamic technique engaged to test for the persistency of mortality rate is the Arellano and Bond [61] two-step difference generalized method of moments (*diff-GMM*) which corrects for endogeneity, cross-sectional dependence, serial correlation and heteroscedasticity by including instruments that are uncorrelated with the regressors in the underlying routine during estimation. Another argument for engaging dynamic panel data modelling is due to the potentially endogenous estimators of the OLS technique which may be biased upwards. This is because the presence of the lagged dependent variable as a regressor creates an endogeneity problem if the OLS approach is used but which is corrected by deploying the GMM technique. The validity of instruments used determines the consistency of the parameters that emanate from such an estimator. Two specification tests put forward by Arellano and Bond [61] to examine the validity of the instruments is the Hansen statistic and second-order serial correlation *AR*(2). Failure to reject the null hypotheses of *over-identifying restrictions are valid*, and no second-order serial correlation gives credence to the results. This study follows the *xtabond2* algorithm designed by Roodman [62]. The *BLSDV* is the baseline model is used to estimate the full and sub-samples, while the *diff-GMM* approach is used only on the full sample as they are not feasible for sub-sample regressions due to the small number of observations (Note: The number of countries in each income group: high income (2), low income (22), and middle income (23). Since the time span is 15 years, only the low- and middle-income countries meet the criteria for performing sys-GMM and because this will make a comparative analysis impossible, this approach is dropped. Hence, only the *BLSDV* approach is used for the comparative income group analysis).

## 4. Results and discussions

This section presents the empirical outcomes of the investigation on whether income per capita exerts a mediating influence on the link between mortality rate, environmental degradation and renewable energy in Sub-Saharan Africa. The sequence of these findings is presented in two parts. The first part dwells on the variables employed in the models, Tables 2 and 3 showcase the descriptive characteristics and pairwise correlation analysis of the variables. The second part details the empirical outcomes. For instance, Table 4 presents results from bootstrap LSDV technique: columns [1] to [4] present the findings of the main models while columns [5] to [8] show the robustness checks when renewable electricity consumption is included as a supplementary control variable. Income groups analyses are displayed in Table 5. Results for the dynamic GMM analysis are presented in Table 6. Finally, Table 7 presents findings from combined robustness checks using (i) quadratic specification of health expenditures and (ii) testing if the impact of renewable energy on morality rate is conditioned on income status. Detailed explanation and discussions are taken in turns.

**Table 2:** Summary statistics.

Variables	*Full Sample*				*High Income*			
	Mean	Std. Dev.	Min	Max	Mean	Std. Dev.	Min	Max
MINF	55.634	22.63	7.8	128	12.757	0.658	11.8	14
MU5	84.033	38.028	9.1	203.6	14.682	0.646	13.8	16
CO2PC	1.076	1.972	0.021	10.428	4.583	1.926	2.684	8.669
REN	64.853	26.598	0.354	97.422	7.042	6.648	0.354	17.737
PC	2540.306	3400.456	208.075	20532.95	10384.66	2348.564	6463.923	14962.38
HEXPC	112.604	142.418	6.621	791.657	488.933	163.988	197.675	791.657
SECF	47.087	27.078	3.74	123.606	88.014	7.598	74.322	100.828
INFL	6.716	7.965	-60.496	63.293	5.451	8.381	-2.405	36.965
BSAN	35.068	23.174	4.556	100	96.459	2.513	92.139	100
URB	41.112	16.479	9.375	89.741	47.797	6.668	40.766	57.119
Variables	*Low Income*				*Middle Income*			
	Mean	Std. Dev.	Min	Max	Mean	Std. Dev.	Min	Max
MINF	64.384	20.186	27	128	50.993	20.075	7.800	101.300
MU5	99.946	34.434	35.300	203.600	74.842	32.842	9.100	166.800
CO2PC	0.136	0.089	0.021	0.445	1.646	2.307	0.154	10.428
REN	82.884	10.727	51.509	97.422	52.633	24.103	5.352	92.961
PC	617.654	288.902	208.075	1900.093	3569.07	3553.156	725.576	20532.95
HEXPC	35.484	25.463	6.621	193.793	150.934	136.022	24.149	597.359
SECF	29.044	11.813	3.740	53.076	60.328	26.295	17.865	123.606
INFL	8.245	9.008	-27.787	63.293	5.558	6.711	-60.496	32.378
BSAN	22.299	13.339	4.556	66.574	41.858	19.894	10.808	98.751
URB	31.726	11.932	9.375	61.931	49.291	16.105	21.675	89.741

Note: MINF = infant mortality rates; MU5 = under-5 mortality rate; CO2PC = carbon emissions per capita; RENEC = renewable energy per capita; PC = GDP per capita (constant 2010); HEXPC = health expenditure per capita; SECF = female secondary school enrolment; INFL = inflation rate; BSAN = access to basic sanitation; URB = urban population.

Source: Authors’ Computations

**Table 3:** Pairwise correlation analysis.

Variables	[1]	[2]	[3]	[4]	[5]	[6]	[7]	[8]	[9]	[10]
(1) lnMINF	1.000									
(2) lnMU5	0.991***	1.000								
(3) lnCO2PC	-0.548***	-0.572***	1.000							
(4) lnREN	0.614***	0.635***	-0.718***	1.000						
(5) lnPC	-0.531***	-0.550***	0.925***	-0.712***	1.000					
(6) lnHEXPC	-0.546***	-0.575***	0.871***	-0.674***	0.911***	1.000				
(7) lnSECF	-0.704***	-0.746***	0.765***	-0.547***	0.703***	0.745***	1.000			
(8) INFL	0.086**	0.08*	-0.155***	0.062	-0.146***	-0.122***	-0.040	1.000		
(9) lnBSAN	-0.535***	-0.558***	0.641***	-0.585***	0.691***	0.715***	0.625***	-0.123***	1.000	
(10) lnURB	-0.212***	-0.248***	0.626***	-0.395***	0.599***	0.486***	0.517***	-0.129***	0.341***	1.000

Note: ***, **, and * denote statistical significance at the 1%, 5%, and 10% levels, respectively; ln = natural logarithm; MINF = infant mortality rates; MU5 = under-5 mortality rate; CO2PC = carbon emissions per capita; RENEC = renewable energy per capita; PC = GDP per capita (constant 2010); HEXPC = health expenditure per capita; SECF = female secondary school enrolment; INFL = inflation rate; BSAN = access to basic sanitation; URB = urban population

Source: Authors’ Computations

**Table 4:** Bootstrap least squares dummy variables results, full sample.

Variables	Main Models				Robustness Models			
	Infant Mortality		Under 5 Mortality		Infant Mortality		Under 5 Mortality	
	[1]	[2]	[3]	[4]	[5]	[6]	[7]	[8]
lnCO2PC	0.067	0.115***	0.053	0.099**	0.150**	0.190***	0.135**	0.174***
	(1.35)	(2.86)	(0.87)	(2.28)	(2.57)	(4.00)	(2.29)	(2.93)
lnREN	0.245***	2.528***	0.262***	2.467***	0.336**	2.738***	0.402***	2.788***
	(6.43)	(8.19)	(5.60)	(6.77)	(2.38)	(4.51)	(3.04)	(6.05)
lnPC	-0.248***	0.780***	-0.217**	0.776***	-0.253***	0.772**	-0.223**	0.794***
	(-2.61)	(5.07)	(-2.35)	(4.76)	(-2.70)	(2.54)	(-2.45)	(3.57)
lnREN*PC		-0.245***		-0.239***		-0.259***		-0.258***
		(-7.47)		(-6.21)		(-3.57)		(-5.09)
lnHEXPC	0.233***	0.216***	0.264***	0.248***	0.290***	0.319***	0.337***	0.366***
	(2.92)	(3.52)	(3.38)	(3.83)	(4.44)	(4.42)	(3.80)	(4.49)
lnSECF	-0.511***	-0.485***	-0.663***	-0.637***	-0.632***	-0.551***	-0.794***	-0.713***
	(-10.05)	(-11.05)	(-10.81)	(-11.56)	(-8.22)	(-9.36)	(-9.51)	(-9.60)
INFL	0.002	0.001	0.001	0.001	-0.001	-0.0004	-0.001	-0.001
	(1.02)	(0.68)	(0.67)	(0.39)	(-0.33)	(-0.19)	(-0.40)	(-0.31)
lnBSAN	-0.1265***	-0.1202***	-0.1666***	-0.1605***	-0.1175***	-0.1138***	-0.1684***	-0.1648***
	(-3.80)	(-4.13)	(-4.36)	(-4.05)	(-3.33)	(-3.49)	(-3.73)	(-3.64)
lnURB	0.153**	0.237***	0.134**	0.215***	0.174***	0.268***	0.148**	0.242***
	(2.42)	(4.66)	(2.01)	(3.15)	(3.46)	(5.12)	(2.50)	(4.00)
lnREL					0.093***	0.070***	0.076***	0.053**
					(3.81)	(4.17)	(3.16)	(2.55)
Low income	0.184	0.236	0.293*	0.344**	0.349*	0.344**	0.378**	0.373**
	(1.15)	(1.62)	(1.84)	(2.16)	(1.71)	(2.08)	(2.27)	(2.11)
Middle income	0.317***	0.495***	0.445***	0.617***	0.391**	0.498***	0.457***	0.564***
	(2.62)	(4.65)	(3.44)	(5.83)	(2.22)	(3.51)	(3.35)	(4.46)
Constant	28.189	19.611	36.908*	28.628	14.923	12.54	20.759	18.392
	(1.49)	(1.15)	(1.68)	(1.21)	(0.67)	(0.64)	(1.04)	(0.88)
Year Dummies	Yes	Yes	Yes	Yes	Yes	Yes	Yes	Yes
Replications	50	50	50	50	50	50	50	50
No. of Obs.	303	303	303	303	261	261	261	261
R-Squared	0.724	0.759	0.764	0.787	0.732	0.767	0.766	0.790
Wald Statistic	936.260***	1904.200***	1453.990***	2154.920***	1469.400***	1751.280***	1140.100***	2423.420***

Note: ***, **, and * denote statistical significance at the 1%, 5%, and 10% levels, respectively; *z* statistics in (); ln = natural logarithm; MINF = infant mortality rates; MU5 = under-5 mortality rate; CO2PC = carbon emissions per capita; REN = renewable energy per capita; PC = GDP per capita (constant 2010); HEXPC = health expenditure per capita; SECF = female secondary school enrolment; INFL = inflation rate; BSAN = access to basic sanitation; URB = urban population; REL = renewable electricity. Models estimated with bootstrapped standard errors.

Source: Authors’ Computations

**Table 5:** Income groups results (dep. Vars: Infant mortality/under-5 mortality).

Variables	Infant Mortality			Under5 Mortality		
	High Income	Low Income	Middle Income	High Income	Low Income	Middle Income
	[1]	[2]	[3]	[4]	[5]	[6]
lnCO2PC	-0.009	-0.050	0.178**	-0.043**	-0.063*	0.171**
	(-1.21)	(-1.47)	(2.37)	(-8.22)	(-1.67)	(2.20)
lnREN	0.942***	1.237	2.418***	1.036***	-0.836	2.800***
	(19.55)	(0.98)	(4.56)	(28.56)	(-0.57)	(5.44)
lnPC	-0.380***	0.474	0.710***	-0.428***	-0.534	0.854***
	(-22.51)	(0.56)	(2.65)	(-39.77)	(-0.55)	(3.48)
lnREN*PC	-0.092***	-0.146	-0.239***	-0.103***	0.092	-0.276***
	(-18.40)	(-0.79)	(-3.54)	(-27.35)	(0.43)	(-4.31)
lnHEXPC	0.133***	0.093	0.441***	0.144***	0.056	0.511***
	(20.17)	(1.36)	(4.46)	(30.97)	(0.78)	(4.69)
lnSECF	0.074*	-0.361***	-0.818***	0.129***	-0.520***	-0.977***
	(3.78)	(-7.92)	(-10.11)	(13.35)	(-9.94)	(-10.71)
INFL	0.001**	0.0003	-0.004	0.001**	-0.001	-0.004
	(8.71)	(0.23)	(-0.94)	(9.29)	(-0.69)	(-0.89)
lnBSAN	9.317**	-0.002	-0.447***	12.791***	-0.019	-0.501***
	(8.23)	(-0.06)	(-5.92)	(15.88)	(-0.52)	(-5.86)
lnURB	-0.309	0.470***	-0.194**	-0.687**	0.385***	-0.167*
	(-2.44)	(10.89)	(-2.34)	(-6.96)	(7.90)	(-1.85)
Constant	21.512**	32.544*	-14.493	29.960***	45.216**	-2.701
	(6.94)	(1.85)	(-0.61)	(12.62)	(2.19)	(-0.10)
Year Dummies	Yes	Yes	Yes	Yes	Yes	Yes
No. of Obs.	22	142	139	22	142	139
R-Squared	1.000	0.648	0.759	1.000	0.679	0.780
F-Statistic	7466.364***	29.242***	24.125***	.	24.302***	32.359***

Note: ***, **, and * denote statistical significance at the 1%, 5%, and 10% levels, respectively; *t* statistics in (); ln = natural logarithm; MINF = infant mortality rates; MU5 = under-5 mortality rate; CO2PC = carbon emissions per capita; REN = renewable energy per capita; PC = GDP per capita (constant 2010); HEXPC = health expenditure per capita; SECF = female secondary school enrolment; INFL = inflation rate; BSAN = access to basic

**Table 6:** 2-step difference GMM results, full sample.

Variables	Main Models				Robustness Models			
	Infant Mortality		Under 5 Mortality		Infant Mortality		Under 5 Mortality	
	[1]	[2]	[3]	[4]	[5]	[6]	[7]	[8]
L.lnMINF/L.lnMU5	0.673***	0.585**	0.960***	0.957***	0.611**	0.721***	0.938***	0.936***
	(3.00)	(2.20)	(26.34)	(23.83)	(2.29)	(4.49)	(40.34)	(20.64)
lnCO2PC	0.003	0.003	0.007**	0.009***	0.005	0.006	0.009**	0.009**
	(0.44)	(0.43)	(2.13)	(3.20)	(0.68)	(0.76)	(2.65)	(2.44)
lnREN	0.023**	0.132**	0.048	0.091	0.027	0.072	0.046	0.048
	(2.56)	(2.31)	(1.59)	(0.69)	(1.07)	(0.24)	(1.35)	(0.26)
lnPC	0.016	0.062*	0.006	0.038	0.034	0.041	0.02	0.019
	(0.68)	(2.02)	(0.24)	(0.45)	(1.07)	(0.22)	(0.76)	(0.17)
lnREN*PC		-0.013**		-0.005		-0.004		-0.0001
		(-2.04)		(-0.29)		(-0.09)		(-0.00)
lnHEXPC	-0.005	-0.006	-0.011	-0.013*	-0.003	-0.003	-0.014	-0.014
	(-1.37)	(-1.67)	(-1.45)	(-2.02)	(-0.47)	(-0.43)	(-1.48)	(-1.46)
lnSECF	-0.022**	-0.024*	-0.003	-0.004	-0.020	-0.016	-0.002	-0.002
	(-2.10)	(-1.88)	(-0.52)	(-0.62)	(-1.49)	(-1.03)	(-0.19)	(-0.22)
INFL	-0.000	-0.000	-0.000	-0.000	-0.000	-0.000	-0.000	-0.000
	(-0.25)	(-0.15)	(-0.32)	(-0.16)	(0.20)	(0.55)	(-0.33)	(-0.28)
lnBSAN	0.007	0.015	0.004	-0.001	0.048	0.031	-0.003	-0.002
	(0.25)	(0.51)	(0.24)	(-0.03)	(0.51)	(0.48)	(-0.12)	(-0.09)
lnURB	0.019	0.023	-0.063	-0.026	0.121	0.073	-0.027	-0.024
	(0.17)	(0.16)	(-0.66)	(-0.25)	(0.48)	(0.33)	(-0.30)	(-0.27)
lnREL					0.0003	0.001	0.007	0.008
					(0.03)	(0.15)	(0.69)	(0.81)
Year Dummies	Yes	Yes	Yes	Yes	Yes	Yes	Yes	Yes
No. of Obs.	215	215	206	206	184	184	182	182
F Statistic	492.992***	295.735***	2156.093***	1626.645***	218.410***	319.076***	20916.701***	17800.214***
Instruments/Groups	27/36	28/36	30/35	31/35	29/31	28/30	31/30	32/30
AR(2)/Hansen	0.155/0.084	0.144/0.171	0.384/0.160	0.389/0.120	0.096/0.063	0.112/0.100	0.365/0.280	0.491/0.805

Note: ***, **, and * denote statistical significance at the 1%, 5%, and 10% levels, respectively; ln = natural logarithm; MINF = infant mortality rates; MU5 = under-5 mortality rate; CO2PC = carbon emissions per capita; REN = renewable energy per capita; PC = GDP per capita (constant 2010); HEXPC = health expenditure per capita; SECF = female secondary school enrolment; INFL = inflation rate; BSAN = access to basic sanitation; URB = urban population; REL = renewable electricity. Source: Authors’ Computations

**Table 7:** Robustness checks on lnHEXPC and lnREN (full and income groups).

Variables	Infant Mortality Rate				Under-5 Mortality Rate				lnMINF	lnMU5
	Full	High	Low	Middle	Full	High	Low	Middle		
	[1]	[2]	[3]	[4]	[5]	[6]	[7]	[8]	[9]	[10]
lnCO2PC	0.0669	0.0321	-0.0465	0.238***	0.0535	0.000652	-0.0609	0.237***	0.170***	0.150**
	(1.298)	(1.809)	(-1.404)	(3.237)	(1.223)	(0.0392)	(-1.595)	(3.028)	(4.251)	(2.554)
lnREN	0.279***	0.0840**	0.292	0.587***	0.297***	0.0828**	-0.224	0.707***	0.107***	0.106***
	(5.638)	(5.142)	(1.601)	(4.602)	(4.571)	(5.027)	(-1.114)	(5.202)	(6.406)	(4.877)
lnPC	-0.271***	-0.294*	-0.162*	-0.180	-0.240**	-0.330*	-0.122	-0.167	-0.174*	-0.145*
	(-2.639)	(-3.686)	(-1.917)	(-1.573)	(-2.554)	(-3.919)	(-1.306)	(-1.351)	(-1.915)	(-1.853)
lnHEXPC	-0.269*	-0.810	-0.231	-0.599	-0.237	-1.086	0.0221	-1.098**	0.169**	0.195***
	(-1.770)	(-0.679)	(-1.086)	(-1.184)	(-1.417)	(-0.882)	(0.0867)	(-2.020)	(2.339)	(3.304)
lnHEXPCSQ	0.0598***	0.0740	0.0445*	0.102*	0.0597**	0.0977	0.00431	0.159***		
	(3.415)	(0.733)	(1.694)	(1.915)	(2.575)	(0.937)	(0.134)	(2.792)		
lnSECF	-0.546***	0.0447	-0.371***	-0.759***	-0.697***	0.105	-0.523***	-0.892***	-0.444***	-0.595***
	(-9.777)	(0.875)	(-7.935)	(-6.877)	(-10.90)	(1.949)	(-9.752)	(-7.494)	(-7.144)	(-8.190)
INFL	0.00176	0.000257	-0.000114	-0.00296	0.00127	1.62e-05	-0.00139	-0.00310	-0.000307	-0.00130
	(0.782)	(0.525)	(-0.0882)	(-0.800)	(0.521)	(0.0318)	(-0.925)	(-0.777)	(-0.221)	(-0.634)
lnBSAN	-0.103***	2.846	0.0118	-0.415***	-0.143***	6.737	-0.0191	-0.451***	-0.0821***	-0.121***
	(-2.849)	(0.450)	(0.344)	(-5.185)	(-3.365)	(1.064)	(-0.470)	(-5.279)	(-2.609)	(-3.515)
lnURB	0.187***	0.391	0.464***	-0.132	0.168**	-0.0706	0.385***	-0.0847	0.181***	0.137**
	(2.800)	(0.410)	(10.72)	(-1.357)	(2.431)	(-0.0737)	(7.676)	(-0.788)	(3.302)	(2.517)
Low income	0.178				0.287				-0.873	0.953
	(1.156)				(1.208)				(-1.025)	(1.181)
Middle income	0.368***				0.496***				-1.359***	-1.356***
	(3.391)				(2.780)				(-3.740)	(-3.456)
lnREN*Low Income									0.481**	0.0939
									(2.264)	(0.475)
lnREN*Middle Income									0.608***	0.650***
									(6.270)	(6.544)
Constant	23.05	11.85	33.88**	6.590	31.78*	23.62	41.49**	21.65	38.76**	48.78***
	(1.168)	(0.509)	(2.152)	(0.295)	(1.779)	(1.005)	(2.214)	(0.842)	(2.160)	(2.709)
Year Dummies	Yes	Yes	Yes	Yes	Yes	Yes	Yes	Yes	Yes	Yes
Observations	303	22	142	139	303	22	142	139	303	303
R-squared	0.732	0.999	0.651	0.750	0.770	0.998	0.679	0.779	0.774	0.810
Wald/F-Statistic	1107	1700	27.93	24.14	1697	515.6	20.70	30.17	10801	13322

Note: ***, **, and * denote statistical significance at the 1%, 5%, and 10% levels, respectively; *z* statistics in (); ln = natural logarithm; MINF = infant mortality rates; MU5 = under-5 mortality rate; CO2PC = carbon emissions per capita; REN = renewable energy per capita; PC = GDP per capita (constant 2010); HEXPC = health expenditure per capita; SECF = female secondary school enrolment; INFL = inflation rate; BSAN = access to basic sanitation; URB = urban population. Models estimated with bootstrapped standard errors. Source: Authors’ Computations

### 4.1. Preliminary analysis

Discussions are limited to the dependent variables and key explanatory variables. From Table 2, infant mortality has an average mean value of 55.63 for the full sample employed in this study. This indicates that sampled countries record an average of 56 deaths per 1000 births within the period under review. However, this figure is not evenly distributed across income groups. Statistics show that the average infant mortality is lowest in high-income countries (12.76) relative to other income groups with 64.34 and 50.99 respectively.

Child mortality follows a similar pattern with an average of 84.03 for the full sample. High income countries record the lowest average (14.68) relative to other income groups with 99.95 and 74.84, respectively. Statistics reveal that carbon emission is more concentrated in high and middle-income countries. On average, the two income groups recorded 4.58 and 1.64, respectively, as against the full sample average of 1.07 metric tons. This gives the indication that carbon emission is higher in high- and medium-income countries relative to low-income countries in Sub-Saharan Africa. Also, the average renewable energy is 64.85 for the full sample, 7.04 for high-income; 52.63 for middle income, and 82.88 for low- income countries. This appears surprising because it is expected that high-income countries embrace more renewable energy sources because of their level of technological innovations and advancement. Similar observations were also noticed for the percentage of renewable electricity in relation to the total electricity consumed during the period. Lastly, the average real per capita income is US$2,540.31 and the standard deviation of 3400.47 shows that the countries are widely dispersed from the sample average. For instance, the average per income across the income groups are US$10,384.66, US$617.65 and US$3,569.07, respectively. Table 3 shows the degree of association among the variables. All the variables, with the exception of inflation, are significantly correlated with the dependent variables. More importantly, infant and child mortality rates exhibit significant inverse correlation with all other variables.

### 4.2. Empirical outcomes–Full sample

This study employs the bootstrap least squares dummy variables (for static model) and two-step difference GMM (for dynamic model). Controlling for yearly variation and similar to Amuka, Asogwa, Ugwuanyi, Omeje, and Onyechi [63], results from Table 4 columns [1] and [3] reveal that the coefficient of carbon emission per capita, though positive, is not significantly associated with infant and under-5 mortality rates. The positive coefficient, notwithstanding, signals the likelihood of contributing to worsening the health of infants and under-5 children [2, 64, 65]. However, *controlling* for the interaction of per capita income with renewable energy (columns [2] and [4]) significantly alters the effect of emissions on mortality rates such that it is associated with increasing the latter by 0.12 and 0.10 percent, on average, *ceteris* paribus (Balani, 2016; Issaoui, Toumi, & Touili, 2015; Matthew, Osabohien, Fagbeminiyi, & Fasina, 2018) [66–68]. The reason may be adduced to the fact that the externalities associated with carbon emission are unfriendly and capable of producing harmful and poisonous substances into the environment. Hence, infants and children may not have a strong immunity to combat the inhalation of harmful substances. Therefore, deaths within the first 60 months of birth are inevitable. The findings from this study are in tandem with the works of Shobande (2020) [8] which noted that infant mortality is significantly influenced by the level of carbon emitted per capita.

Also, across all model specifications, renewable energy (columns [1] and [3]) has a significant positive and detrimental effect on mortality rates. The plausible explanatiom maybe because SSA countries which are mostly low-income developing economies have less health facilities powered by renewable energy sources majorly due to the huge capital outlay.

The statistically significant coeficients of per capita income (columns [1] and [3]) reveal that income exerts an ameliorating effect on mortality rates from between -0.248 and -0.217 percent. On the moderating effect of per capita income (columns [2] and [4]), we find that per capita income exert a statistically significant negative moderating effect, -0.245 and -0.239 percent, respectively. The *conditonal* effect of renewable energy is therefore computed as [2.5282 –(0.2479*$\bar{\ln PC}$] for infant mortality and [2.4665 –(0.2393*$\bar{\ln PC}$)] for under-5 mortality, respectively (Note: For simplicity, lnPC is evaluated at its mean values). Given the negative coefficient of the interaction term, these outcomes imply that in the presence of increased per capita income the devastating effect of renewable energy on mortality rates diminishes. However, the magnitude of influence will depend on the values of real per capita income.

For the control variables, contrary to expectations, a percentage change in health expenditures exacebates mortality rates between 0.22 and 0.26 percent. The plausible explanation for this outcome which contradicts Nkalu and Edeme [69] and Boachie, Ramu, and Põlajeva [70] is that paltry allocations to healthcare in most SSA countries may be driving high mortalities. As expected, urbanization puts pressure on available health facilities culminating in high morality rates from about 0.13 to 0.24 percent (Shobande, 2020) [8]. Similarly, female education and access to basic sanitation significantly contributes to reducing mortality rates [8, 9, 71, 72]. The income group dummies reveal that middle-income countries have significantly higher morality rates than high income country (base dummy) whose coefficient is captured by the constant term. The intercept of middle-income countries are positive and statistically significant across all models. Cursory observation reveals that under-5 mortality rate is significantly higher in low-income countries relative to high-income countries. Aside the significant coefficients for carbon emissions, the outcomes of the robustness checks are not significantly different from those of the main results. The models pass the the basic diagnostics. The goodness-of-fit shows that the regressors cause between 72.4% and 79% of the variation in mortality rates while the Wald statistic indicates that the regressors are jointly significant in predicting mortality rates.

### 4.3. Empirical outcomes–Income groups

The sample is further divided into three to observe if the results hold across income groups (Note: We are unable to perform robustness checks using renewable electricity variable due to few data points for high income countries having only 2 countries)) and the outcomes are presented in Table 5. Carbon emission is negatively associated with infant and under-5 mortalities in middle-income countries at the 5% significance level while it is positively associated with under-5 mortalities in high- and low-income countries, respectively.

Also, renewable energy consumption is positively associated in high- and middle-income countries while real income per capita shows a negative association with infant and under-5 mortalities in high income countries but positive association in middle-income countries. Our study shows that real per capita income is a significant channel to reducing mortality rate in SSA. Following Eq [3], the total effects of renewable energy on infant and under-5 mortality rate when real per capita income is computed at its mean values (Note: Mean values of lnPC for high, low and middle-income countries are: 9.223, 6.338, and 7.821, respectively) are as follows:

$$\frac{\partial \ln MINF}{\partial \ln REN}=0.942-(0.092*9.223)=0.09\left(\text{High}\;\text{income}\;\text{countries,}\;\text{infant}\;\text{mortality}\;\text{rate}\right)$$

$$\frac{\partial \ln MINF}{\partial \ln REN}=2.418-(0.239*7.821)=0.55\left(\text{Middle-income}\;\text{countries,}\;\text{infant}\;\text{mortality}\;\text{rate}\right)$$

$$\frac{\partial \ln MU5}{\partial \ln REN}=1.036-(0.103*9.223)=0.086\left(\text{High}\;\text{income}\;\text{countries,}\;\text{under-5}\;\text{mortality}\;\text{rate}\right)$$

$$\frac{\partial \ln MU5}{\partial \ln REN}=2.800-(0.276*7.821)=0.64\left(\text{High}\;\text{income}\;\text{countries,}\;\text{under-5}\;\text{mortality}\;\text{rate}\right)$$

These results vividly show that real per capita income is essential in reducing the positive association of renewable energy on infant and under-5 mortality rates from 0.942% to 0.09%, 2.42% to 0.55%, 1.04% to 0.09% and 2.8% to 0.64% for high and middle-income countries, respectively. This is a novel and significant contribution to the health-environment literature.

On the control variables, health expenditure per capita is positively associated with infant and under-5 mortalities in high and medium-income groups. Moreover, inflation is a positive predictor of infant and under-5 mortalities in high-income countries. Female education reduces mortality rates in low and middle-income countries but aggravates in high-income countries. Similarly, access to basic sanitation shows a reducing effect on mortality rates in middle-income countries while it increases mortalities in high-income countries. Finally, urbanization has a significant negative effect on infant and under-5 mortalities in high and middle-income groups, but its effect is positive in low-income countries. Overall, we observe that these outcomes are significantly different across income groups.

### 4.4. Empirical outcomes–Dynamic analysis

Controlling for heteroscedasticity, omitted variables and serial correlation, we estimate a dynamic model to test for the persistency of mortality rates. The results displayed in Table 6 reveal that infant and under-5 mortalities are persistent in SSA [8, 9, 73]. This is reflected in the positive and statistically significant values of the lagged dependent variables in both primary and robustness models. Explicitly, a percentage change in the previous year’s mortality rates leads to between 0.67 and 0.96 percent increase in infant and under-5 mortalities.

Similar to Table 4, carbon emissions is significantly associated with high under-5 mortality rate while renewable energy is positively associated with infant mortality. The findings further suggest that the interaction of renewable energy consumption and income per capita is negatively associated with infant mortality, howbeit marginally. On model diagnostics, the F-statistics show that the regressors are jointly significant in explaining mortality rates given their level of significance at 1%. The *AR*(2) statistics further suggest no evidence of second-order serial correlation while the Hansen statistics confirm the validity of the instruments. Hence, the findings obtained from the augmented regression models can be used for inferences.

### 4.5. Robustness checks

Since our results from the effect of health expenditure and renewable energy are counter-intuitive, we probe further by: (1) engaging a quadratic analysis to test the second-order effect of health expenditure on mortality rate and observe if there are significant differences across the full sample and income groups. The results are displayed in columns [1]–[8] of Table 7. (2) We test if the effect of renewable energy on mortality rate is contingent on the level of economic development of a country. To achieve this, renewable energy is interacted with each income group dummy using high income group as the base dummy variable and the results are shown in columns [9] and [10] of Table 7. Starting with the results from the quadratic specification, we find a significant U-shaped effect of health expenditures from the full sample for infant mortality rate whereas the results across the income groups are mixed. While the first-order effect is negative but statistically not significant for all income groups, the second-order effect is positive and significant at the 10% level for low- and middle-income countries. In column 8, the U-shaped relationship between health expenditures and under-5 mortality is evident only in middle-income countries while the results from other samples are either not significant or inconclusive. Overall, health expenditure is positively associated with infant and under-5 mortality rates in SSA countries and the earlier reasoning provided for such counter-intuitive outcome subsists.

From column [9], the intercept of the model (38.76) represents the average infant mortality rate in high-income countries. The results show that infant mortality rate in low- and middle-income countries are lower than those of high-income countries by -0.87% and -1.36%, respectively. Also, the coefficient of renewable energy (0.107) for high-income countries suggests that renewable energy is positively linked to infant mortality rate by 0.11%. The association is stronger in low-income countries by 0.59% (that is, 0.11 + 0.48) and in middle-income countries by 0.72% (that is, 0.11 + 0.61). Analogous deductions for under-5 mortality rate. In conclusion, we submit that variations in the effect of renewable energy on mortality rate in contingent on the income classification of a country.

## 5. Conclusion and policy recommendations

This paper takes a multi-dimensional approach towards the actualisation of the 2030 United Nations Sustainable Development Goals 3 and 11 by examining the effect of carbon emissions, renewable energy and real per capita income on mortality rate in Sub-Saharan Africa (SSA). Using an unbalanced panel data on 47 SSA countries and a battery of econometric techniques, specifically, this study focused on infant and under-5 infant mortality rates because mortality rates are development determinants and crucial to assessing human capital development. We also considered five key questions related to carbon emissions, use of renewable energy, including per capita GDP. In addition, we accounted for the contribution of other factors, female secondary school enrolment, health expenditure per capita, urbanization, access to basic sanitation and consumer prices. Consistent findings from a multi-analytical approach using bootstrapped least squares dummy variables and difference generalised method of moments reveal that: (1) both carbon emissions and renewable energy are significantly associated with high mortality rates; (2) per capita income is significantly associated with reducing mortality rates; (3) per capita income weakens the devastating effect of renewable energy on mortality rates, (4) both infant and under-5 mortality rates are persistent at the 1% significance levels, and (5) the health-environment-energy-income dynamics differ across income groups. In other words, with respect to the presence of renewable energy sources, we find that despite the availability of environmentally-friendly energy sources, there is a significant increase in infant and under-5 mortality rates. Furthermore, this study submits that the interaction of renewable energy and income dampens the positive effect of renewable energy on mortality rates and supports the argument that income levels lessen the extent of mortalities. Hence, per capita income is a crucial determinant of mortality rates.

Policy recommendations are not far-fetched. First, there is need for policy makers to initiate interventions to tackle health challenges of babies and children in SSA; secondly, need to extend the factors considered as health outcomes determinants and actively address environmental factors; thirdly, by giving cognizance to environmental factors, policymakers and development practitioners will be able to come up with solutions that ensure that most babies and children do not die in their early ages but live and reach their potential which enables them to contribute to the continued advancement of the region. Giving available data, the mediating effect of carbon emissions on the energy-health dynamics may be taken up in future research.

## Supporting information

S1 File (XLSX)
